# Postmenopausal Presentation of Granulosa Cell Tumor: A Case Report

**DOI:** 10.7759/cureus.77175

**Published:** 2025-01-09

**Authors:** Risha Patel, Anuja Bhalerao, Ojas Bondre

**Affiliations:** 1 Department of Obstetrics and Gynaecology, NKP Salve Institute of Medical Sciences and Research Centre, Nagpur, IND

**Keywords:** granulosa cell tumor, malignant neoplasm, omentectomy, postmenopausal, salpingo-oophorectomy

## Abstract

The granulosa cells found in the ovarian follicles give rise to malignant neoplasms known as granulosa cell tumors (GCTs). The majority of the diagnoses are reported in Stage I and surgery is the main intervention for the adult form, which is frequently reported. Hence, the current report presents a case of GCT in postmenopausal women who reported the chief complaints of pain in the abdomen on the left side, bloating since three to four days, and the presence of spotting in the previous month. On general examination, a mass of 18 weeks was palpated which was firm, tender, and mobile. Moreover, Magnetic Resonance Imaging (MRI) showed the presence of a solid cystic pelvic-abdominal lesion that was suggestive of malignant epithelial neoplastic etiology of ovarian origin. Based on the findings, staging laparotomy was planned and commenced along with abdominal hysterectomy, bilateral salpingo-oophorectomy, omentectomy, and peritoneal biopsy with iliac lymphadenectomy. The histopathology (HPE) report revealed the presence of GCT in the left ovary (stage IIIA2). Hence, despite the generally favorable prognosis for early-stage GCTs, vigilance in long-term follow-up is crucial due to the potential for late recurrence. To execute appropriate and timely intervention and customized treatment regimens, this case highlights the significance of multidisciplinary coordination in the diagnosis and treatment of ovarian tumors that are uncommon.

## Introduction

Of all ovarian sex cord-stromal tumors, malignant neoplasms originating from granulosa cells of the ovarian follicles constitute around 10% [1]. Granulosa cell tumors (GCTs) are classified into two groups: juvenile type, which involves 5% of cases, and adult type, which includes 95% of cases. The adult type, which peaks between the ages of 50 and 55 years, is most frequently observed in postmenopausal women [2]. The majority are restricted to the ovary and are considered as stage I. Metastases of the lymph nodes are extremely uncommon, with over 95% demonstrating unilateral involvement, and are limited to the ovaries. They are typically encapsulated, smooth, and lobulated and can have areas of hemorrhage and necrosis. Based on the amount of lipids and the degree of luteinization, the surfaces can be yellow or tan. Additionally, depending on the fibromatous content, they can also be hard or soft. The tumors that are more luteinized represent more orange or yellow color [3]. The estrogen, anti-Müllerian hormone (AMH), and inhibin B synthesis by tumors are linked to the hyperestrogenism observed in GCT patients [4].

The adult form is characterized by slow evolution and late recurrences with rate of 54%. Based on the morphological information and histological evaluation, the diagnosis is determined [5-7], stage I in the majority of the cases, for which surgery represents the primary intervention. The standard surgical treatment includes bilateral salpingo-oophorectomy, total abdominal hysterectomy, and thorough surgical staging that includes peritoneal biopsies and omentectomy [5]. Patients with advanced stages or untreatable recurring illnesses are considered for radiotherapy and/or chemotherapy [4]. For stage III GCT, standard chemotherapy regimens like BEP (bleomycin, etoposide, cisplatin) serve as a choice of treatment. Hence, the present case report aims to highlight clinical examination, diagnostic assessment, and therapeutic intervention performed for GCTs.

## Case presentation

Patient information

A 59-year-old female married for 40 years reported to the Obstetrics and Gynaecology outpatient department with primary complaints of pain in the abdomen on the left side and bloating for three to four days and the presence of spotting in the previous month. The menstrual history described that the patient was postmenopausal for 13 years. Additionally, the patient reported a history of loss of appetite and weight for six months. The obstetric history of the patient reported parity three and three live births (P3L3); all were full-term normal deliveries along with tubal ligation.

Clinical examination

The patient was vitally stable. The breasts were soft and non-tender bilaterally. On per abdominal examination, a mass of 18 weeks was palpated, which was firm, tender, and mobile. On per vaginal examination, a mobile mass of 18 weeks was noted, and the cervix was found to be deviated to the left side. The uterus was retroverted and the mobile firm mass was felt through all the fornices. Moreover, a groove was present between the mass and the uterus, making the differential diagnosis of dysgerminoma and mucinous tumor. Following this, the laboratory investigations revealed that the tumor marker CA-125 value was 23.16 U/mL, lactate dehydrogenase, which is used as a marker for tissue damage, lay in the normal range with a value of 150 U/L, alpha-fetoprotein (AFP) tumor marker value 8.28 ng/mL was found to be within normal limits, and beta-human chorionic gonadotropin (B-HCG) levels were within normal limits < 2 mIU/mL indicating negative pregnancy test but the elevated level of inhibin B of 500 pg/mL confirmed the diagnosis of GCT.

Diagnostic assessment

**Figure 1:**
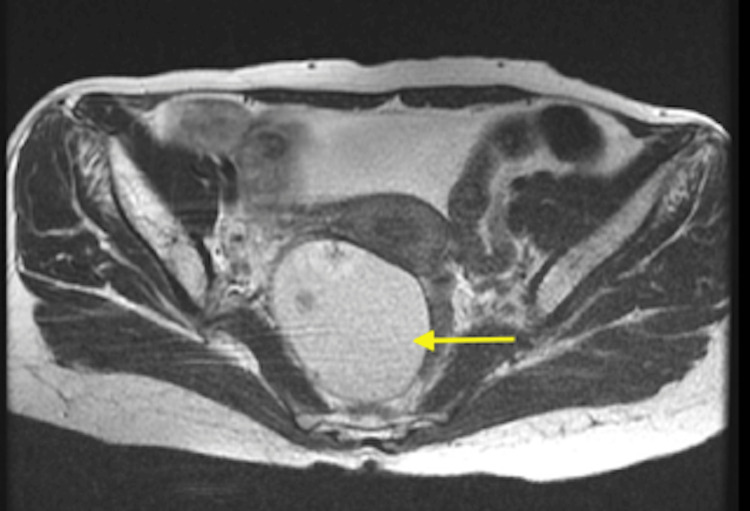
T2 MRI image of the patient The image shows a cystic pelvic-abdominal lesion of 15 x 12 x 11 cm marked with a yellow arrow MRI = Magnetic Resonance Imaging

**Figure 2:**
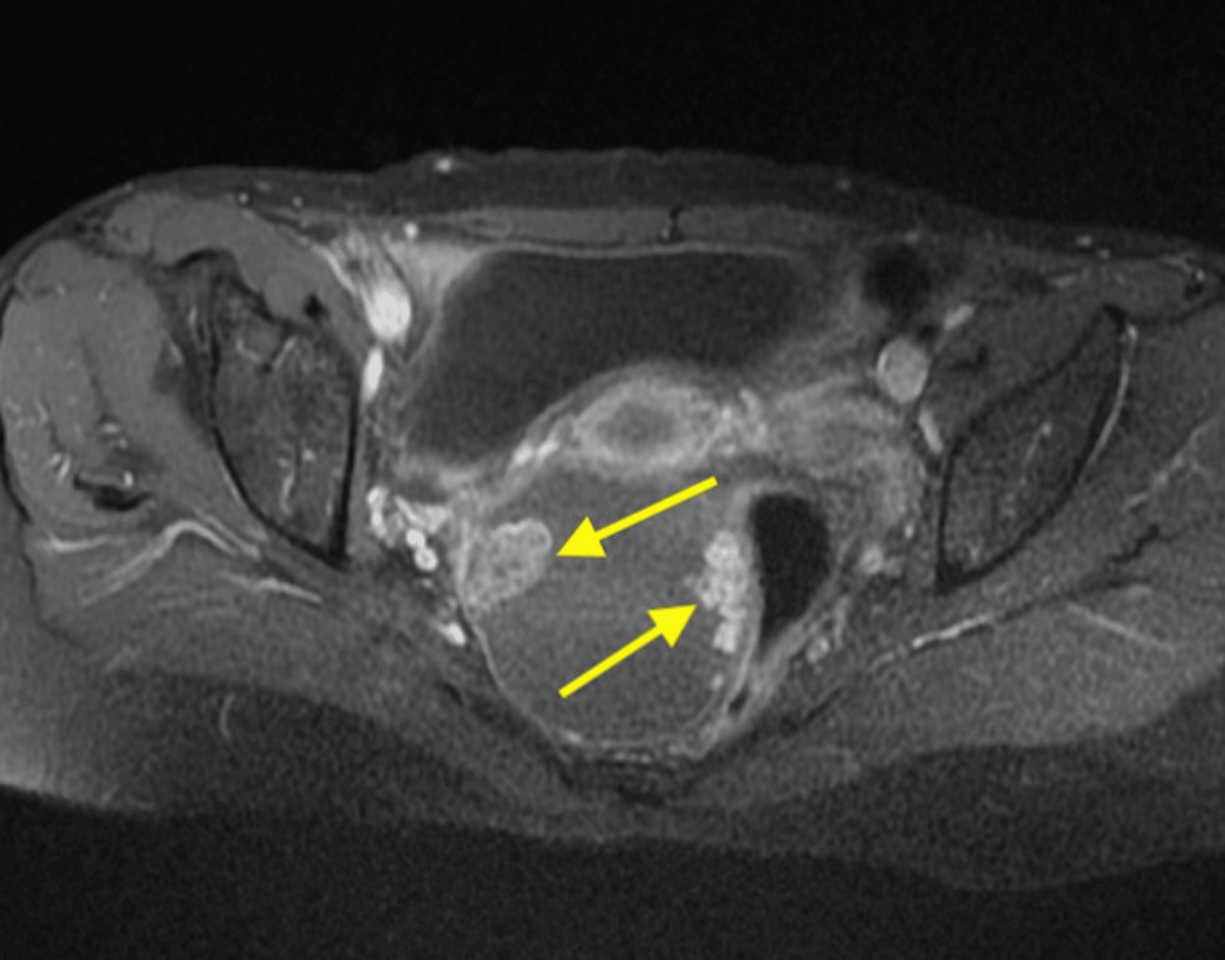
The post-contrast MRI image The image shows the solid pelvic-abdominal lesion. MRI = Magnetic Resonance Imaging

Transvaginal ultrasound and Magnetic Resonance Imaging (MRI) were performed for diagnostic assessment. The transvaginal ultrasound demonstrated a solid cystic lesion of size 15 x 12 x 11 cm in the left adnexa. The T2 image, illustrated in Figure 1, showed a cystic lesion, and the post-contrast image, shown in Figure 2, showed a solid pelvic-abdominal lesion, suggesting a malignant epithelial neoplastic etiology of ovarian origin. Also, in the fine needle aspiration cytology test, the diagnosis inclined in favor of adult GCT based on the presence of “coffee bean” nuclei with a proliferation of small round cells and Call-Exner bodies. Additionally, a small biopsy was suggestive of bleeding endometrium with proliferative glands.

Therapeutic intervention

**Figure 3:**
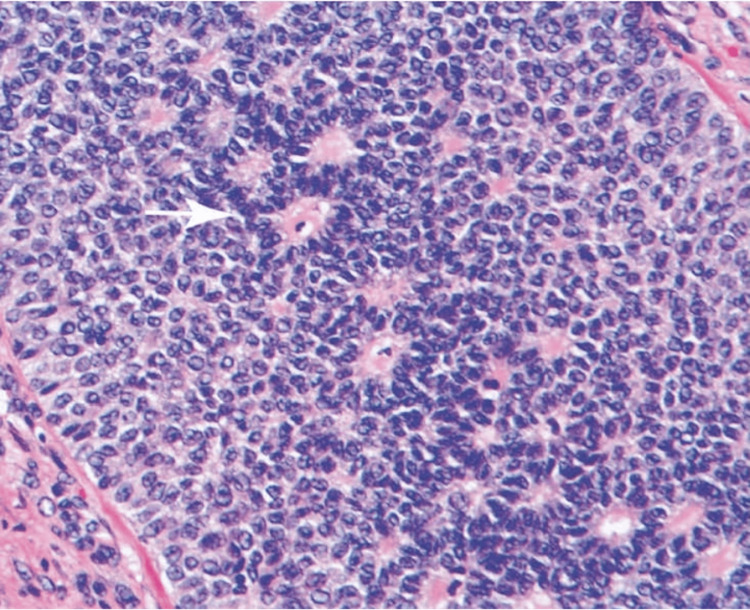
Histopathological examination image Granulosa tumor cells highlighting uniform, pale, scanty cytoplasm, round to oval nuclei with nuclear grooves (coffee bean nuclei) and Call-Exner bodies.

As a part of the diagnosis and therapeutic intervention, surgical staging laparotomy with total abdominal hysterectomy, bilateral salpingo-oophorectomy, omentectomy, and peritoneal biopsy with iliac lymphadenectomy was performed. The specimens were then directed for histopathological examination (HPE). The HPE report revealed the presence of GCT in the left ovary (FIGO stage IIIA2), as illustrated in Figure 3, tumor deposits on the omentum, and unremarkable left and right iliac lymph nodes. For stage IIIA2 GCT, a standard chemotherapy regimen like BEP (bleomycin, etoposide, cisplatin) had been involved in chemotherapy. Moreover, the peritoneal fluid cytology showed positive microscopic peritoneal metastatic cells which was suggestive of stage IIIA2 GCT.

## Discussion

The granulosa cell layer of the ovarian follicles increases in number in reaction to growing gonadotropin levels in the blood and decreases with an increase in testosterone levels. The theca and granulosa cells normally produce estradiol and envelop the growing oocyte by forming the cellular stroma around it. The hypothalamus and anterior pituitary detect excess estradiol, which suppresses the release of follicle-stimulating hormone (FSH), gonadotropin-releasing hormone, and luteinizing hormone (LH) [8]. FSH-induced aromatization in follicles and FSH binding are enhanced when activin stimulates granulosa cells. Additionally, this strengthens the effect of LH on the theca cells, which increases androgen production. Inhibins A and B and AMH are also produced by granulosa cells. When FSH is stimulated, the body produces Inhibin B, which gives direction to the pituitary to decrease FSH production. Abdominal pain, postmenopausal bleeding, and irregular uterine bleeding are frequently caused by excess estrogen that is released by the tumor [9]. Similarly, in the present study, the patient reported abdominal pain and bleeding which corresponds with the above findings. Excess estrogen from GCTs increases endometrial hyperplasia and raises the risk of breast cancer in women with uterus [10].

The hallmark of histological analysis involves a low mitotic rate along with Call-Exner bodies and “Coffee-bean” nuclei. Call-Exner bodies resemble ovarian follicles and are glandular in appearance. The nuclei of "coffee bean" are round, grooved longitudinally, and pale [1]. In the present case report, the diagnosis in favor of adult GCT was oriented based on the presence of “coffee bean” nuclei with proliferation of small round cells and Call-Exner bodies.

The recurrence rates in Stage I are low (5.4%), however, in Stages III and IV the rates increase to 21.1% and 40%, respectively [11,12]. Patients diagnosed with more advanced stages and with stage IC usually require adjuvant chemotherapy. For GCT tumors in the early stages, unilateral salpingo-oophorectomy is the primary intervention performed [13]. However, in the present case, the stage of the GCT tumor was stage IIIA2 referring to a more advanced stage.

In terms of clinical characteristics, the capacity of GCT to manufacture estrogens results in a variety of clinical symptoms [7]. According to Lee et al., the development of symptoms such as abdominal bloating and pain (10%-20%), and irregular vaginal bleeding (45%), are caused due to the synthesis of estrogen [14]. Whereas, in post-menopausal women, the clinical presentation consists of unilateral ovarian mass with abnormal bleeding, and rarely in the early stages ascites may be present (10%) [7]. These are solid tumors that can be either hard or soft, based on the proportion of fibrothecomatous stroma and neoplastic cells present [7]. Similar findings were reported in the present study demonstrating left-side abdominal pain, bloating, presence of a firm, mobile, solid cystic pelvic-abdominal lesion that was suggestive of malignant epithelial neoplastic etiology of ovarian origin.

Differentiating adult GCTs from carcinoid tumors, adenocarcinomas, and undifferentiated carcinomas can be challenging. The prognosis for each of these malignancies is quite variable. The appearance of the nuclei is their characteristic or distinguishing aspect. GCTs are characterized by their grooved, uniformly pale, angular, or oval nuclei, which resemble coffee beans. Undifferentiated carcinomas frequently have hyperchromatic, unevenly sized, and shaped nuclei that are not grooved. Multiple mitotic figures and nuclear atypia are also uncommon in granulosa tumors but are more common in undifferentiated carcinomas [7].

A crucial aspect of the treatment of adult GCT is early diagnosis along with surgical management. Moreover, comprehensive genomic research might offer directions toward the etiology, pathology, and possible courses of treatment. Future research on adult GCTs is essentially required to determine biomarkers for response monitoring and to assess the effectiveness of alternative treatment agents in addition to endocrine therapy for long-term use.

## Conclusions

This case highlights the clinical and pathological complexity of adult granulosa cell tumors (GCT), an ovarian neoplasm that typically presents in postmenopausal women. The patient, diagnosed at FIGO stage IIIA2, underscores the indolent yet potentially aggressive nature of GCTs, particularly in advanced stages. Surgical management, including total abdominal hysterectomy, bilateral salpingo-oophorectomy, and comprehensive staging, remains the cornerstone of treatment for optimal outcomes. Despite the generally favorable prognosis for early-stage GCTs, vigilance in long-term follow-up is crucial due to the potential for late recurrence. This case emphasizes the importance of multidisciplinary collaboration including geneticists or oncologists in the diagnosis and management of ovarian tumors to ensure timely intervention and tailored therapeutic strategies. Further research is needed to better understand the pathogenesis, improve early detection methods and reliable tumor markers, and explore targeted therapies for recurrent GCTs.
